# Pandemic (H1N1) 2009 Outbreak on Pig Farm, Argentina

**DOI:** 10.3201/eid1602.091230

**Published:** 2010-02

**Authors:** Ariel Pereda, Javier Cappuccio, María A. Quiroga, Elsa Baumeister, Lucas Insarralde, Mariela Ibar, Ramón Sanguinetti, Maria L. Cannilla, Débora Franzese, Oscar E. Escobar Cabrera, Maria I. Craig, Agustina Rimondi, Mariana Machuca, Rosa T. Debenedetti, Carlos Zenobi, Leonardo Barral, Rodrigo Balzano, Santiago Capalbo, Adriana Risso, Carlos J. Perfumo

**Affiliations:** Instituto Nacional de Tecnología Agropecuaria, Castelar, Buenos Aires, Argentina (A. Pereda, M.I. Craig, A. Rimondi); Universidad Nacional de La Plata, La Plata, Buenos Aires (J. Cappuccio, M.A. Quiroga, L. Insarralde, M. Ibar, M. Machuca, C.J. Perfumo); Instituto Nacional de Enfermedades Infecciosas–Administración Nacional de Laboratorios e Institutos de Salud, Buenos Aires (E. Baumeister); Servicio Nacional de Sanidad Animal, Martínez, Buenos Aires (R. Sanguinetti, M.L. Cannilla, D. Franzese, O.E. Escobar Cabrera, R.T. Debenedetti, C. Zenobi, L. Barral, R. Balzano); private veterinary practice, Buenos Aires (S. Capalbo, A. Risso); 1 These authors contributed equally to this article.

**Keywords:** Pigs, pandemic H1N1 2009, epidemiology, pathology, immunohistochemistry, real-time RT-PCR, Argentina, influenza, viruses, expedited, dispatch

## Abstract

In June–July 2009, an outbreak of pandemic (H1N1) 2009 infection occurred on a pig farm in Argentina. Molecular analysis indicated that the virus was genetically related to the pandemic (H1N1) 2009 influenza virus strain. The outbreak presumably resulted from direct human-to-pig transmission.

Swine influenza viruses (SIVs) occur worldwide; they usually cause asymptomatic infection but occasionally cause outbreaks of varying intensity (*1*,^2^). In North America, the landscape of swine influenza has changed substantially since the late 1990s, when human (H3N2) viruses were first isolated from swine and triple reassortant viruses carrying influenza genes (TRIG) of avian, swine, and human origin and showing great reassortment flexibility emerged concurrently. TRIGs of influenza subtypes H3N2, H1N1, H3N1, and H1N2 appear to be circulating in swine in North America. Coincidentally, 1 of these TRIG viruses led to emergence of the pandemic (H1N1) 2009 virus (*3*). Testing by the Canadian Food Inspection Agency identified pandemic (H1N1) 2009 virus in a swine herd in Alberta (*4*). The pigs may have been exposed to the virus by a farm worker who returned from Mexico with influenza-like symptoms (*5*). Affected pigs showed clinical signs of infection with SIV (*5*).

During the past influenza season (April–September 2009) in the Southern Hemisphere, pandemic (H1N1) 2009 virus predominated in humans and accounted for >90% of influenza cases in humans in Argentina. In Argentina, seroepidemiologic analyses of pigs on 17 farms showed that ≈41% of pigs had antibodies against H1 and H3 (*6*). Vaccines against SIV are not licensed for use in Argentina.

We describe the epidemiology, clinical outcome, pathology, and persistence of pandemic (H1N1) 2009 infection on a pig farm 103 km from Buenos Aires. Molecular analysis indicated that the virus was genetically related to the pandemic (H1N1) 2009 virus strain and indicated no evidence of further reassortment with any other influenza strains. The outbreak presumably resulted from direct human-to-pig transmission at the peak of the pandemic in Argentina.

## The Study

The pig farm, a closed farrow-to-finish 1-site operation in Buenos Aires province, had 519 sows. Pig death rate from farrowing to slaughter remained at 9.5% before and after the outbreak. Ten days before the outbreak, the farm manager and his wife reported clinical signs of influenza in themselves.

On June 15, 2009, nursery barn pigs >40 days of age showed clinical signs, e.g., cough, dyspnea, fever, nasal discharge, and inappetence. Illness affected 30% of nursery pigs. On June 17, pigs in 8 growing and fattening barns showed similar clinical signs; 4,000 (15%) pigs were affected). Clinical signs lasted for 1 week; on June 22, resolution of clinical signs was evident, with cough index <2% in growing and fattening.

At the onset of the outbreak, 5 clinically affected pigs were submitted for postmortem examination. Tissue samples from nose, trachea, and lungs were fixed in 10% buffered formalin and processed for routine histopathologic examination. We conducted immunohistochemical (IHC) analysis using antinucleoprotein influenza A monoclonal antibody, isotype MSIg2a (Chemicon International Inc., Temecula, CA, USA) according to Vincent et al. (*7*). In addition, we obtained 30 serum samples from nursery, growing, and fattening pigs.

All pigs had cranioventral lung consolidation on 5%–60% of the total surface. Two of 5 pigs had distinctive, scattered, dark-red foci of lobular consolidation (chessboard-like) (Figure, panel A). In 3 pigs, we observed serofibrinous polyserositis.

**Figure:**
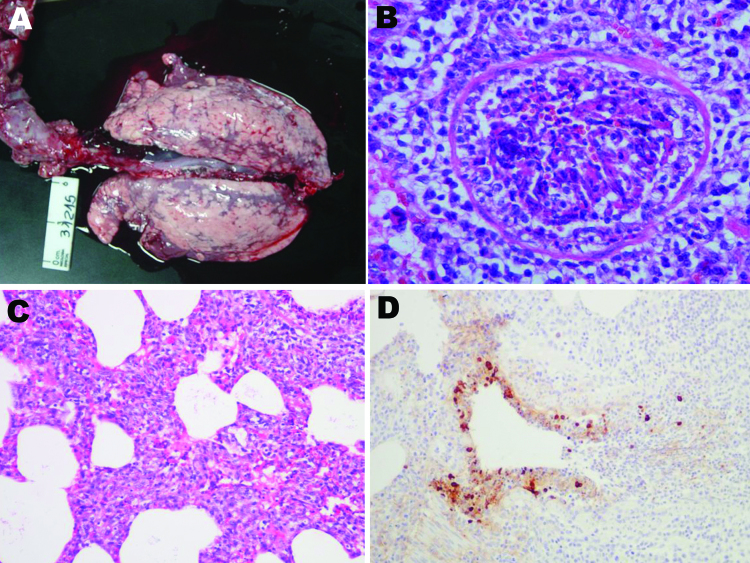
Postmortem samples from clinically affected pigs, Argentina, 2009. A) Macroscopic lung lesions with distinctive scattered dark-red foci of lobular consolidation (chessboard-like) in all lobes. B) Severe necrotizing bronchiolitis with partially denuded epithelia. Hematoxylin-eosin stain; original magnification ×400. C) General view of the alveolar walls showing moderate interstitial thickening by leukocytes. Hematoxin-eosin stain; original magnification ×200. D) Bronchioli. Positive-labeled nuclei and cytoplasm of bronchial epithelial cells and mononuclear cells in the alveoli. 3,3′-diaminobenzidine counterstain with hematoxylin stain; original magnification ×200.

Four pigs had severe necrotizing bronchiolitis. Small and medium bronchioles were plugged with neutrophils cellular debris and exudates. Affected airways were denuded or lined with flat epithelium (Figure, panel B). The adjacent alveolar walls were infiltrated and thickened in a lobular fashion by mononuclear cells (Figure, panel C). In other pigs, the alveolar lumen was filled with macrophages, neutrophils, and fibrinous exudates. We observed a positive IHC reaction in bronchiolar epithelia only, which showed severe inflammatory changes (Figure, panel D).

We processed 5 bronchial swabs and lung samples from postmortem-examined pigs for real-time reverse transcription–PCR (rRT-PCR) and for virus isolation in MDCK cells. Viral RNA was extracted from swab suspension and from culture supernatant (Total RNA Isolation Kit; Applied Biosystems, Foster City, CA, USA), and viral cDNA was synthesized. We tested the cDNA for influenza type A by rRT-PCR directed to the matrix (M) gene, as previously reported (*8*), and tested for pandemic (H1N1) 2009 virus by rRT-PCR using the protocol released by the Centers for Disease Control and Prevention (CDC) on April 30, 2009 (revision 1) (www.who.int/csr/resources/publications/swineflu/realtimeptpcr/en/index.html).

Next, we conducted a cross-sectional study of 60 nasal swabs from pigs of 3 categories (i.e., nursery, growing, fattening) on June 25, July 2, July 10, and July 31. All these samples were negative. Also, 120 bronchial swabs were sampled at slaughter on June 30, July 3, and July 7; 1 sample from June 30 and 5 from July 3 were positive, but thereafter, all bronchial swabs were negative. A serologic survey was conducted of 214 fattening pigs on June 30 and every week thereafter by using ELISA (HerdChek Swine Influenza H1N1 Antibody Kit; IDEXX Laboratories, Westbrook, ME, USA).

All postmortem samples were positive for subtype (H1N1) according to both rRT-PCR protocols, and cycle threshold values ranged from 16 to 27 cycles according to the CDC protocol. Nasal samples from pigs in the cross-sectional study were negative. However, 5% of the bronchial swabs sampled from clinically healthy pigs at slaughter were positive according to both rRT-PCR protocols. At the onset of the outbreak, all pig serum samples were negative for swine (H1N1) influenza virus. However, 98% of serum samples from slaughter pigs were positive 15 days later.

Cytopathic effects in MDCK cells were noted on first passage at 72 h postinoculation, and this virus was characterized by sequencing as pandemic (H1N1) 2009. All genome segments were fully amplified by RT-PCR (*9*) and directly sequenced from the isolate in MDCK. Regions corresponding to the entire open reading frame of hemagglutinin (HA), nucleoprotein, neuraminidase (NA), M1, and nonstructural and partial polymerase base (PB) 2 (nt 580–1140), PB1 (nt 390–990), and polymerase acid **(**PA**)** (nt 720–1230) segments were obtained by using appropriate set of primers and submitted to GenBank (accession nos. CY044256–CY044260 and CY047749–CY047751). High (>99.99%) nucleotide identity was found between these sequences and pandemic strain A/California/04/2009 (H1N1) and A/swine/Alberta/OTH-33-8/2009 (H1N1) (Table).

**Table:** Nucleotide identity percentage of swine influenza viruses in a study of a pandemic (H1N1) 2009 outbreak at a pig farm, Argentina, June–July 2009

Genome segment*	A/California/04/ 2009 (H1N1)	A/swine/Alberta/OTH-33-8/ 2009 (H1N1)
1 (PB2)	100	99.994
2 (PB1)	100	99.998
3 (PA)	100	99.998
4 (HA)	99.997	99.994
5 (NP)	99.996	99.993
6 (NA)	99.998	99.995
7 (M1)	99.997	99.996
8 (NS)	99.999	99.994

*PB, polymerase base; PA, polymerase acid; HA, hemagglutinin; NP, nucleoprotein; NA, neuraminidase; M, matrix; NS, nonstructural protein.

## Conclusions

Young pigs are susceptible to pandemic (H1N1) 2009 as reported in Canada (*4*,^5^). Recently, experimental infections showed that the pandemic (H1N1) 2009 virus induced mild clinical signs, virus shedding, and gross and histopathologic lesions similar to those caused by SIV (*10*,*11*). Successful cross-species transmission usually requires a period of adaptation to the new host (*12*). However, phylogenetic analyses of pandemic (H1N1) 2009 virus showed that the HA and NA glycoproteins arose from the classical swine (H1N1) and avian-like Eurasian swine (H1N1) lineages, respectively (*3*), which might explain the high susceptibility and transmissibility among pigs.

From an epidemiologic standpoint, the 25%–30% morbidity rate with few deaths agreed with the clinical presentation of SIV (H1N1) (*1**,**13*) and was similar to the rates found in the outbreak in Canada (*4*,^5^). Clinical signs lasted 1 week. However, 5% of the bronchial swabs taken 15–18 days after onset of the outbreak from slaughter-weight pigs showed positive results. Virus shedding, detected by rRT-PCR, from nasopharyngeal samples peaked at 4 days postinoculation and ceased at ≈11 days postinoculation.

Epithelial cell necrosis, sloughing, and neutrophil infiltration with total or partial obstruction of bronchioles is the hallmark of SIV infection (*14*) and was distinctive for influenza A virus (H1N1). However, IHC results were strongly positive for virus antigen in only those bronchioles that showed severe bronchiolitis. Our findings from field observation confirm findings from experimental studies with pandemic (H1N1) 2009 virus (*11*).

The National Veterinary Services Laboratory has established a farm biosecurity program to survey the infection. The program includes movement restrictions and clinical surveillance of farms within 3-km of the index case. No other farm had clinically ill pigs. The farm was allowed to move animals to slaughter after the nasal swabs of those animals tested negative by rRT-PCR with the CDC protocol.

The suspected human source of infection was not confirmed. This outbreak supports the belief that influenza cross-species transmission may occur between pigs and humans and vice versa.

## References

[1] Olsen CW, Brown IH, Easterday BC, Van Reeth K. Swine influenza. In: Straw BL, Zimmerman JJ, D’Alleire S, Taylor DJ, editors. Diseases of swine. 9th ed. Ames (IA): Blackwell Publishing; 2006. p. 469–82.

[2] Yoon KJ, Janke BH. Swine influenza: etiology, epidemiology, and diagnosis. In: Morilla A, Yoon KJ, Zimmerman JJ, editors. Trends in emerging viral infections of swine. Ames (IA): Blackwell Publishing; 2002. p. 23–8.

[3] Peiris JS, Poon LL, Guan Y. Emergence of a novel swine-origin influenza A virus (S-OIV) H1N1 virus in humans. J Clin Virol. 2009;45:169–73. 10.1016/j.jcv.2009.06.006 19540800 PMC4894826

[4] Canadian Food Inspection Agency. A/H1N1 influenza, Canada. Immediate notification. 02/05/2009 [cited 2009 May 2]. http://www.oie.int/wahis/public.php?page=single_report&pop=1&reportid=8065

[5] Canadian Food Inspection Agency. A/H1N1 influenza, Canada. Follow-up report no. 1. 11/06/2009 [cited 2009 Jun 11]. http://www.oie.int/wahis/public.php?page=single_report&pop=1&reportid=8180

[6] Teodoroff TA, Pecoraro MR, Baumeister E, Janke BH, Machuca M, Cappuccio J, Serological and immunohistochemical studies of influenza virus in fattening pigs in Argentina. In: Martelli P, Cavirani S, Lavazza A, editors. Proceedings of the 4th International Symposium on Emerging and Re-emerging Pig Diseases; 2003 June 29–Jul 2; Rome, Italy. Parma (Italy): University of Parma; 2003. p. 262–63.

[7] Vincent LL, Janke BH, Paul PS, Halbour PG. A monoclonal-antibody–based immunohistochemical method for the detection of swine influenza virus in formalin-fixed, paraffin-embedded tissues. J Vet Diagn Invest. 1997;9:191–5. 9211240 10.1177/104063879700900214

[8] Spackman E, Senne DA, Myers TJ, Bulaga LL, Garber LP, Perdue ML, Development of a real-time reverse transcriptase PCR assay for type A influenza virus of the avian H5 and H7 hemagglutinin subtypes. J Clin Microbiol. 2002;40:3256–60. 10.1128/JCM.40.9.3256-3260.2002 12202562 PMC130722

[9] Hoffmann E, Stech J, Guan Y, Webster RG, Perez DR. Universal primer set for the full-length amplification of all influenza A viruses. Arch Virol. 2001;146:2275–89. 10.1007/s007050170002 11811679

[10] Brookes SM, Irvine RM, Nunez A, Clifford D, Essen S, Brown IH, Influenza A (H1N1) infection in pigs. Vet Rec. 2009;164:760–1. 19525527 10.1136/vr.164.24.760

[11] Lange E, Kalthoff D, Blohm U, Teifke JP, Breithaupt A, Maresch C, Pathogenesis and transmission of the novel swine origin influenza virus A/H1N1 after experimental infection of pigs. J Gen Virol. 2009;90:2119–23. 10.1099/vir.0.014480-0 19592456

[12] Olsen CW, Busch GM, Bateman AC, Landolt G, Karasin AI. Swine influenza pathogenesis: interspecies transmission of influenza viruses through pigs. In: Markowska-Daniel I, editor. Proceedings 5th International Symposium on Emerging and Re-emerging Pig Diseases. Krakow, Poland; 2007 Jun 24–27. Krakow (Poland): Jagiellonian University. p. 225–8.

[13] Brown IH. The epidemiology and evolution of influenza viruses in pigs. Vet Microbiol. 2000;74:29–46. 10.1016/S0378-1135(00)00164-4 10799776

[14] Jung T, Choi C, Chae C. Localization of swine influenza virus in naturally infected pigs. Vet Pathol. 2002;39:10–6. 10.1354/vp.39-1-10 12102201

